# Complete genome sequence of *Granulicella tundricola* type strain MP5ACTX9^T^, an *Acidobacteria* from tundra soil

**DOI:** 10.4056/sigs.4648353

**Published:** 2013-12-05

**Authors:** Suman R. Rawat, Minna K. Männistö, Valentin Starovoytov, Lynne Goodwin, Matt Nolan, Loren Hauser, Miriam Land, Karen Walston Davenport, Tanja Woyke, Max M. Häggblom

**Affiliations:** 1 Department of Biochemistry and Microbiology, Rutgers, The State University of New Jersey, New Brunswick, New Jersey USA; 2 Finnish Forest Research Institute, Rovaniemi, Finland; 3 Department of Cell Biology and Neuroscience, Rutgers, The State University of New Jersey, Piscataway, New Jersey, USA.; 4 Los Alamos National Laboratory, Bioscience Division, Los Alamos, New Mexico, USA; 5 DOE Joint Genome Institute, Walnut Creek, California, USA; 6 Oak Ridge National Laboratory, Oak Ridge, Tennessee, USA

**Keywords:** cold adapted, acidophile, tundra soil, *Acidobacteria*

## Abstract

*Granulicella tundricola* strain MP5ACTX9^T^ is a novel species of the genus *Granulicella* in subdivision 1 *Acidobacteria*. *G. tundricola* is a predominant member of soil bacterial communities, active at low temperatures and nutrient limiting conditions in Arctic alpine tundra. The organism is a cold-adapted acidophile and a versatile heterotroph that hydrolyzes a suite of sugars and complex polysaccharides. Genome analysis revealed metabolic versatility with genes involved in metabolism and transport of carbohydrates, including gene modules encoding for the carbohydrate-active enzyme (CAZy) families for the breakdown, utilization and biosynthesis of diverse structural and storage polysaccharides such as plant based carbon polymers. The genome of *G. tundricola* strain MP5ACTX9^T^ consists of 4,309,151 bp of a circular chromosome and five mega plasmids with a total genome content of 5,503,984 bp. The genome comprises 4,705 protein-coding genes and 52 RNA genes.

## Introduction

The strain MP5ACTX9^T^ (=ATCC BAA-1859^T^ =DSM 23138^T^) is the type strain of *Granulicella tundricola* [tun.dri.co’la. N.L. n. *tundra,* tundra, a cold treeless region; L. masc. suffix -*cola (from L. n. incola)* dweller; N.L. n. *tundricola* tundra dweller] that was isolated from soil at the Malla Nature Reserve, Kilpisjärvi, Finland; 69°01’N, 20°50’E) and described along with other species of the genus *Granulicella* isolated from tundra soil [1].

*Acidobacteria* is a phylogenetically and physiologically diverse phylum [2,3], the members of which are ubiquitously found in diverse habitats and are abundant in most soil environments [4,5] including Arctic tundra soils [6,7]. *Acidobacteria* are relatively difficult to cultivate, as they have slow growth rates. To date only subdivisions 1, 3, 4, 8, 10 and 23 *Acidobacteria* are defined by taxonomically characterized representatives [8-23] as well as three ‘Candidatus’ taxa [24,25]. The phylogenetic diversity, ubiquity and abundance of this group suggest that they play important ecological roles in soils. The abundance of *Acidobacteria* correlates with soil pH [26,27] and carbon [28,29], with subdivision 1 *Acidobacteria* being most abundant in slightly acidic soils. *Acidobacteria*, including members of the genera *Granulicella* and *Terriglobus*, dominate the acidic tundra heaths of northern Finland [26,30-32]. Using selective isolation techniques we have been able to isolate several slow growing and fastidious strains of *Acidobacteria* [1,11]. On the basis of phylogenetic, phenotypic and chemotaxonomic data, including 16S rRNA, *rpoB* gene sequence similarity and DNA–DNA hybridization, strain MP5ACTX9^T^ was classified as a novel species of the genus *Granulicella* [1]. Here, we summarize the physiological features together with the complete genome sequence, annotation and data analysis of *Granulicella tundricola* strain MP5ACTX9^T^.

## Classification and features

Within the genus *Granulicella*, eight species are described with validly published names: *G. mallensis* MP5ACTX8^T^, *G. tundricola* MP5ACTX9^T^, *G. arctica* MP5ACTX2^T^,*G. sapmiensis* S6CTX5A^T^ isolated from Arctic tundra soil [1] and *G. paludicola* OB1010^T^, *G. paludicola* LCBR1, *G. pectinivorans* TPB6011^T^ ,*G. rosea* TPO1014^T^ ,*G. aggregans* TPB6028^T^ isolated from sphagnum peat bogs [2].

**Figure 1:**
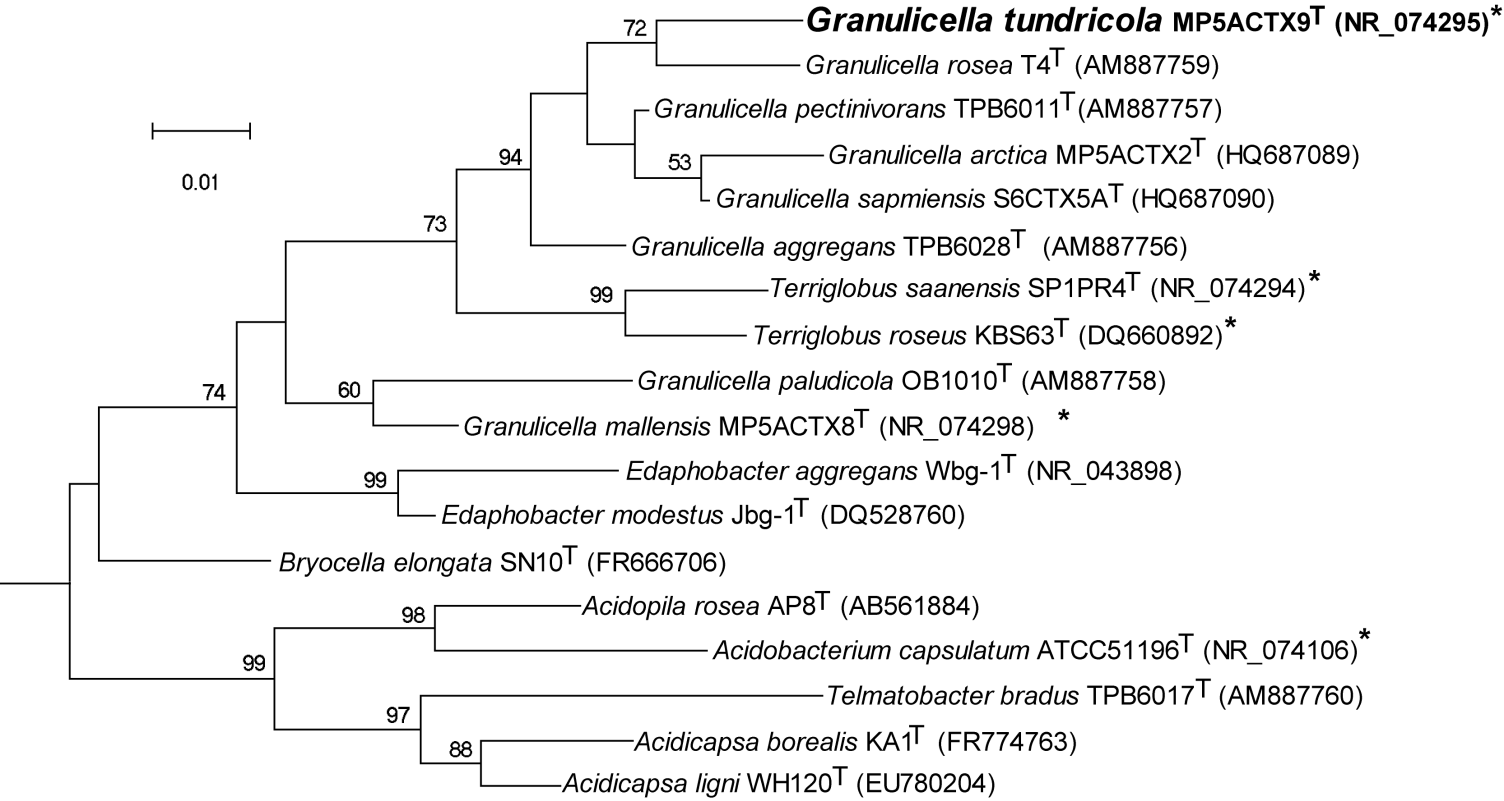
Phylogenetic tree highlighting the position of *G. tundricola* MP5ACTX9^T^ (shown in bold) relative to the other type strains within subdivision1 *Acidobacteria*. The maximum likelihood tree was inferred from 1,361 aligned positions of the 16S rRNA gene sequences and derived based on the Tamura-Nei model using MEGA 5 [42]. Bootstrap values >50 (expressed as percentages of 1,000 replicates) are shown at branch points. Bar: 0.01 substitutions per nucleotide position. The corresponding GenBank accession numbers are displayed in parentheses. Strains whose genomes have been sequenced, are marked with an asterisk; *G. mallensis* MP5ACTX8^T^ (CP003130), *G. tundricola* MP5ACTX9^T^ (CP002480), *T. saanensis* SP1PR4^T^ (CP002467), *T. roseus* KBS63^T^ (CP003379), and *A. capsulatum* ATCC 51196^T^ (CP001472). *Bryobacter aggregatus* MPL3 (AM162405) in SD3 *Acidobacteria* was used as an outgroup.

Strain MP5ACTX9^T^ shares 95.5 - 97.2% 16S rRNA gene identity with tundra soil strains *G. mallensis* MP5ACTX8^T^ (95.5%), *G. arctica* MP5ACTX2^T^ (96.9%), *G. sapmiensis* S6CTX5A^T^ (97.2%) and 95.2 – 97.7% identity with the sphagnum bog strains, *G. pectinivorans* TPB6011^T^ (97.7%), *G. rosea* TPO1014^T^ (97.2%), %), *G. aggregans* TPB6028^T^ (96.8%), *G. paludicola* LCBR1 (95.9%), and *G. paludicola* strain OB1010^T^ (95.3%), which were isolated from sphagnum peat. Phylogenetic analysis based on the 16S rRNA gene of taxonomically classified strains of family *Acidobacteriaceae* placed *G. rosea* type strain T4^T^ (AM887759) as the closest taxonomically classified relative of *G. tundricola* strain MP5ACTX9^T^ (Table 1, Figure 1).

**Table 1:** Classification and general features of *G. tundricola* strain MP5ACTX9^T^

**MIGS ID**	**Property**	**Term**	**Evidence code^a^**
	Classification	Domain *Bacteria*	TAS [33]
		Phylum *Acidobacteria*	TAS [34,35]
		Class *Acidobacteria*	TAS [36,37]
		Order *Acidobacteriales*	TAS [37,38]
		Family *Acidobacteriaceae*	TAS [35,39]
		Genus *Granulicella*	TAS [1,40]
		Species *Granulicella tundricola*	TAS [1]
		Type strain: MP5ACTX9^T^ (ATCC BAA-1859^T^ = DSM 23138^T^)	
	Gram stain	negative	TAS [1]
	Cell shape	rod	TAS [1]
	Motility	non-motile	TAS [1]
	Sporulation	not reported	NAS
	Temperature range	4–28°C	TAS [1]
	Optimum temperature	21–24 °C	TAS [1]
	pH range; Optimum	3.5–6.5; 5	TAS [1]
	Carbon source	D-glucose, maltose, cellobiose, D-fructose, D-galactose, lactose, lactulose, D-mannose, sucrose, trehalose, D-xylose, raffinose, N-acetyl-D-glucosamine, glutamate	TAS [1]
MIGS-6	Habitat	terrestrial, tundra soil	TAS [1]
MIGS-6.3	Salinity	No growth with >1.0% NaCl (w/v)	TAS [1]
MIGS-22	Oxygen requirement	aerobic	TAS [1]
MIGS-15	Biotic relationship	free-living	TAS [1]
MIGS-14	Pathogenicity	non-pathogen	NAS
MIGS-4	Geographic location	Malla Nature Reserve, Arctic-alpine tundra, Finland	TAS [1]
MIGS-5	Sample collection	2006	TAS [1]
MIGS-4.1	Latitude	69°01’N	TAS [1]
MIGS-4.2	Longitude	20°50’E	TAS [1]
MIGS-4.4	Altitude	700 m	TAS [1]

^a^ Evidence codes - IDA: Inferred from Direct Assay; TAS: Traceable Author Statement (i.e., a direct report exists in the literature); NAS: Non-traceable Author Statement (i.e., not directly observed for the living, isolated sample, but based on a generally accepted property for the species, or anecdotal evidence). These evidence codes are from the Gene Ontology project [41].

### Morphology and physiology

**Figure 2:**
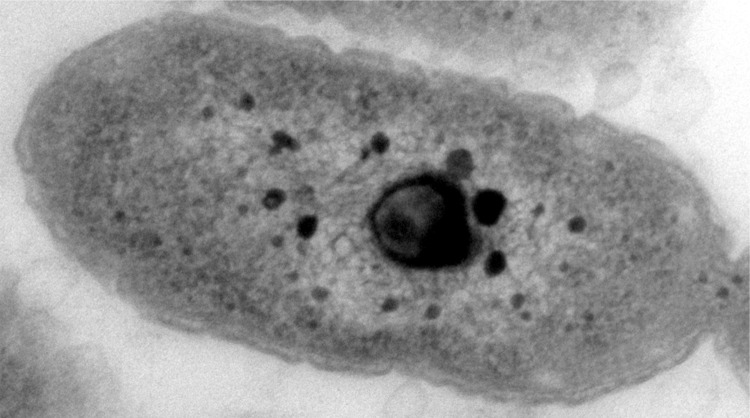
Electron micrograph of *G. tundricola* MP5ACTX9^T^

*G. tundricola* cells are Gram-negative, non-motile, aerobic rods, approximately 0.5 μm wide and 0.5 – 1.8 μm long. Colonies on R2A agar are pink, circular, convex and smooth. Growth occurs at +4 to 28°C and at pH 3.5-6.5 with an optimum at 21-24°C and pH 5 (Fig. 2). Genotypic analyses, including low *rpoB* gene sequence similarity and phenotypic characteristics clearly distinguished strain MP5ACTX9^T^ from other *Granulicella* species/strains, leading us to conclude that MP5ACTX9^T^ represents a novel species of the genus *Granulicella*, for which the name *Granulicella tundricola* sp. nov. was proposed [1].

Strain MP5ACTX9^T^ hydrolyzed complex to simple carbon substrates [1] which include complex polysaccharides like aesculin, pectin, laminarin, starch and pullulan, but not gelatin, cellulose, lichenan, sodium alginate, xylan, chitosan or chitin. Strain MP5ACTX9^T^ also utilized the following sugars as growth substrates: D-glucose, maltose, cellobiose, D-fructose, D-galactose, lactose, lactulose, D-mannose, sucrose, trehalose, D-xylose, raffinose, N-acetyl-D-glucosamine, glutamate and gluconic acid. Enzyme activities reported for the strain MP5ACTX9^T^ include acid phosphatase, esterase (C4 and C8), leucine arylamidase, valine arylamidase, α-chymotrypsin, trypsin, naphthol-AS-BI-phosphohydrolase, α- and β-galactosidases, α- and β-glucosidases, N-acetyl- β-glucosaminidase, β-glucuronidase, α-fucosidase and α-mannosidase but negative for alkaline phosphatase and lipase (C14). Strain MP5ACTX9^T^ is resistant to ampicillin, erythromycin, chloramphenicol, neomycin, streptomycin, tetracycline, gentamicin, bacitracin, polymyxin B and penicillin, but susceptible to rifampicin, kanamycin, lincomycin and novobiocin.

### Chemotaxonomy

The major cellular fatty acids in *G. tundricola* are iso-C_15:0_ (46.4%), C_16:1ω7c_ (35.0%) and C_16:0_ (6.6%). The cellular fatty acid composition of strain MP5ACTX9^T^ was similar to that of other *Granulicella* strains with fatty acids iso-C_15:0_ and C_16:1ω7c_ being most abundant in all strains. Strain MP5ACTX9^T^ contains MK-8 as the major quinone and also contains 4% of MK-7.

## Genome sequencing and annotation

### Genome project history

*G. tundricola* strain MP5ACTX9^T^ was selected for sequencing in 2009 by the DOE Joint Genome Institute (JGI) community sequencing program. The Quality Draft (QD) assembly and annotation were completed on May 24, 2010. The GenBank Date of Release was February 2, 2011. The genome project is deposited in the Genomes On-Line Database (GOLD) [43] and the complete genome sequence of strain MP5ACTX9^T^ is deposited in GenBank (CP002480.1). Table 2 presents the project information and its association with MIGS version 2.0 [44].

**Table 2:** Project information.

**MIGS ID**	**Property**	**Term**
MIGS 31	Finishing quality	Finished
MIGS-28	Libraries used	Three libraries, an Illumina GAii shotgun library (GUIX), a 454 Titanium standard library (GTWG, GWTA) and a paired end 454 (GSUN) library
MIGS 29	Sequencing platforms	454 Titanium standard, 454 Paired End, Illumina
MIGS 31.2	Fold coverage	20×(454), 274X (Illumina)
MIGS 30	Assemblers	Newbler, VELVET, PHRAP
MIGS 32	Gene calling method	ProdigaL, GenePRIMP
	Locus Tag	AciX9
	Genbank ID	CP002480.1
	GenBank Date of Release	February 2, 2011
	GOLD ID	Gc01833
	BIOPROJECT	PRJNA50551, PRJNA47621
	Project relevance	Environmental, Biogeochemical cycling of Carbon, Biotechnological, GEBA

### Growth conditions and genomic DNA extraction

*G. tundricola* MP5ACTX9^T^ was cultivated on R2 medium as previously described [1]. Genomic DNA (gDNA) of high sequencing quality was isolated using a modified CTAB method and evaluated according to the Quality Control (QC) guidelines provided by the DOE Joint Genome Institute [45].

### Genome sequencing and assembly

The finished genome of *G. tundricola* MP5ACTX9^T^ (JGI ID 4088693) was generated at the DOE Joint genome Institute (JGI) using a combination of Illumina [46] and 454 technologies [47]. For this genome we constructed and sequenced an Illumina GAii shotgun library which generated 42,620,699 reads totaling 3239 Mb, a 454 Titanium standard library which generated 146,119 reads and three paired end 454 libraries with an average insert size of 9.3 kb which generated 178,757 reads totaling 154.3 Mb of 454 data. All general aspects of library construction and sequencing performed at the JGI can be found at the JGI website [45]. The 454 Titanium standard data and the 454 paired end data were assembled with Newbler, version 2.3. Illumina sequencing data was assembled with Velvet, version 0.7.63 [48]. The 454 Newbler consensus shreds, the Illumina Velvet consensus shreds and the read pairs in the 454 paired end library were integrated using parallel phrap, version SPS - 4.24 (High Performance Software, LLC) [49]. The software Consed [50] was used in the finishing process. The Phred/Phrap/Consed software package [51] was used for sequence assembly and quality assessment in the subsequent finishing process. Illumina data was used to correct potential base errors and increase consensus quality using the software Polisher developed at JGI (Alla Lapidus, unpublished). Possible misassemblies were corrected using gapResolution (Cliff Han, un-published), Dupfinisher [52] or sequencing cloned bridging PCR fragments with sub-cloning. Gaps between contigs were closed by editing in Consed, by PCR and by Bubble PCR (J-F Cheng, unpublished) primer walks. The final assembly is based on 29.1 Mb of 454 draft data which provides an average 20× coverage of the genome and 975 Mb of Illumina draft data which provides an average 274× coverage of the genome.

### Genome annotation

Genes were identified using Prodigal [53] as part of the Oak Ridge National Laboratory genome annotation pipeline, followed by a round of manual curation using the JGI GenePRIMP pipeline [54]. The predicted CDSs were translated and used to search the National Center for Biotechnology Information (NCBI) non-redundant database, UniProt, TIGRFam, Pfam, PRIAM, KEGG, (COGs) [55,56], and InterPro. These data sources were combined to assert a product description for each predicted protein. Non-coding genes and miscellaneous features were predicted using tRNAscan-SE [57], RNAMMer [58], Rfam [59], TMHMM [60], and signalP [61]. Additional gene prediction analysis and functional annotation were performed within the Integrated Microbial Genomes Expert Review (IMG-ER) platform [62].

## Genome properties

**Figure 3:**
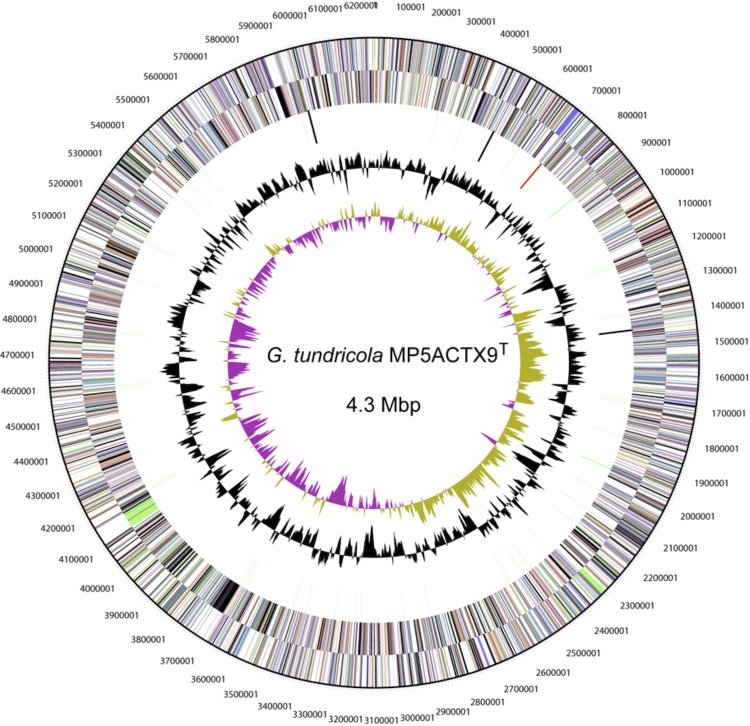
Circular representation of the chromosome of *G. tundricola* MP5ACTX9^T^ displaying relevant genome features. From outside to center; Genes on forward strand (colored by COG categories), genes on reverse strand (colored by COG categories), RNA genes (tRNAs green, rRNAs red, other RNAs black), GC content and GC skew.

**Figure 4:**
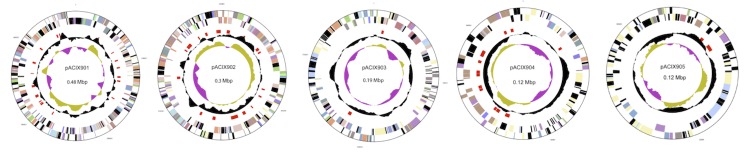
Circular representation of the plasmids of *G. tundricola* MP5ACTX9^T^ displaying relevant genome features. From outside to center; Genes on forward strand (color by COG categories), genes on reverse strand (color by COG categories), RNA genes (tRNAs green, rRNAs red, other RNAs black), GC content and GC skew. Order and size from left to right: pACIX901, 0.48 Mbp; pACIX902, 0.3 Mbp; pACIX903, 0.19 Mbp; pACIX904, 0.12 Mbp; pACIX905, 0.12 Mbp.

The genome is 5,503,984 bp in size, which includes the 4,309,151 bp chromosome and five plasmids pACIX901 (0.48 Mbp); pACIX902 (0.3 Mbp); pACIX903 (0.19 Mbp), pACIX904 (0.12 Mbp) and pACIX905 (0.12 Mbp), with a GC content of 59.9 mol%. There are 52 RNA genes (Figures 3 and 4, and Table 3). Of the 4,758 predicted genes, 4,706 are protein-coding genes (CDSs) and 163 are pseudogenes. Of the total CDSs, 68.8% represent COG functional categories and 27.5% consist of signal peptides. The distribution of genes into COG functional categories is presented in Figure 3 and Table 4, and Table 5.

**Table 3:** Summary of genome: one chromosome and five plasmids

**Label**	**Size (Mb)**	**Topology**	**INSDC identifier**	**RefSeq ID**
Chromosome	4.3	circular	CP002480.1	NC_015064.1
Plasmid pACIX901	0.48	circular	CP002481.1	NC_015057.1
Plasmid pACIX902	0.3	circular	CP002482.1	NC_015065.1
Plasmid pACIX903	0.19	circular	CP002483.1	NC_015058.1
Plasmid pACIX904	0.12	circular	CP002484.1	NC_015059.1
Plasmid pACIX905	0.12	circular	CP002485.1	NC_015060.1

**Table 4:** Genome statistics.

**Attribute**	**Value**	**% of Total**
Genome size (bp)	5,503,984	100
DNA coding (bp)	4,759,459	86.5
DNA G+C (bp)	3,301,098	60.0
DNA scaffolds	6	100
Total genes	4,757	100
Protein coding genes	4,705	98.9
RNA genes	52	1.1
Pseudo genes	163	3.4
Genes in internal clusters	2,395	50.4
Genes with function prediction	2,936	61.7
Genes assigned to COGs	3,259	68.5
Genes with Pfam domains	3,504	73.6
Genes with signal peptides	652	13.7
Genes with transmembrane helices	1,108	23.3
CRISPR repeats	0	-

The total is based on either the size of the genome in base pairs or the protein coding genes in the annotated genome.

**Table 5:** Number of genes associated with general COG functional categories.

**Code**	**Value**	**%age**	**Description**
J	160	4.45	Translation, ribosomal structure and biogenesis
A	2	0.06	RNA processing and modification
K	249	6.93	Transcription
L	222	6.18	Replication, recombination and repair
B	1	0.03	Chromatin structure and dynamics
D	33	0.92	Cell cycle control, Cell division, chromosome partitioning
V	68	1.89	Defense mechanisms
T	212	5.9	Signal transduction mechanisms
M	287	7.98	Cell wall/membrane biogenesis
N	73	2.03	Cell motility
U	123	3.42	Intracellular trafficking and secretion
O	125	3.48	Posttranslational modification, protein turnover, chaperones
C	174	4.84	Energy production and conversion
G	248	6.9	Carbohydrate transport and metabolism
E	234	6.51	Amino acid transport and metabolism
F	68	1.89	Nucleotide transport and metabolism
H	147	4.09	Coenzyme transport and metabolism
I	126	3.5	Lipid transport and metabolism
P	137	3.81	Inorganic ion transport and metabolism
Q	91	2.53	Secondary metabolites biosynthesis, transport and catabolism
R	446	12.41	General function prediction only
S	370	10.29	Function unknown
-	1498	31.49	Not in COGs

The total is based on the total number of protein coding genes in the genome.

## Discussion

*Granulicella tundricola* MP5ACTX9^T^ is a tundra soil strain with a genome consisting of a circular chromosome and five mega plasmids ranging in size from 1.1 x 10^5^ to 4.7 x 10^5^ bp for a total genome size of 5.5 Mbp. The *G. tundricola* genome also contains close to twice as many pseudogenes and a large number of mobile genetic elements as compared to *Granulicella mallensis* and *Terrigobus saanensis*, two other *Acidobacteria* isolated from the same habitat [29]. A large number of genes assigned to COG functional categories for transport and metabolism of carbohydrates (6.9%) and amino acids (6.5%) and involved in cell envelope biogenesis (8%) and transcription (6.9%) were identified. Further genome analysis revealed an abundance of gene modules encoding for functional activities within the carbohydrate-active enzymes (CAZy) families [63,64] involved in breakdown, utilization and biosynthesis of carbohydrates. *G. tundricola* hydrolyzed complex carbon polymers, including CMC, pectin, lichenin, laminarin and starch, and utilized sugars such as cellobiose, D-mannose, D-xylose and D-trehalose. Genome predictions for CDSs encoding for enzymes such as cellulases, pectinases, alginate lyases, trehalase and amylases are in agreement with biochemical activities in strain MP5ACTX9^T^. However, the genome of *G. tundricola* did contain many CDSs encoding for GH18 chitinases although no chitinase activity was detected after 10 day-incubation with chitinazure [29]. In addition, the *G. tundricola* genome contained a cluster of genes in close proximity to the cellulose synthase gene (*bcsAB*), which included cellulase (*bscZ*) (endoglucanase Y) of family GH8, cellulose synthase operon protein (*bcsC*) and a cellulose synthase operon protein (yhjQ) involved in cellulose biosynthesis. We previously reported on a detailed comparative genome analysis of *G. tundricola* MP5ACTX9^T^ with other *Acidobacteria* strains for which finished genomes are available [29]. The data suggests that *G. tundricola* is involved in hydrolysis and utilization of stored carbohydrates and biosynthesis of exopolysaccharides from organic matter and plant based polymers in the soil. Therefore, *G. tundricola* may be central to carbon cycling processes in Arctic and boreal soil ecosystems.

## References

[1] Männistö MK Rawat S Starovoytov V Häggblom MM *Granulicella arctica* sp. nov., *Granulicella mallensis* sp. nov., *Granulicella sapmiensis* sp. nov. and *Granulicella tundricola* sp. nov., novel *Acidobacteria* from tundra soil of Northern Finland. Int J Syst Evol Microbiol 2012; 62:2097-2106 10.1099/ijs.0.031864-0 22058325

[2] Jones RT Robeson MS Lauber CL Hamady M Knight R Fierer N A comprehensive survey of soil acidobacterial diversity using pyrosequencing and clone library analyses. ISME J 2009; 3:442-453 10.1038/ismej.2008.127 19129864 PMC2997719

[3] Barns SM Cain EC Sommerville L Kuske CR *Acidobacteria* phylum sequences in uranium-contaminated subsurface sediments greatly expand the known diversity within the phylum. Appl Environ Microbiol 2007; 73:3113-3116 10.1128/AEM.02012-06 17337544 PMC1892891

[4] Janssen PH Identifying the dominant soil bacterial taxa in libraries of 16S rRNA and 16S rRNA genes. Appl Environ Microbiol 2006; 72:1719-1728 10.1128/AEM.72.3.1719-1728.2006 16517615 PMC1393246

[5] Fierer N Bradford MA Jackson RB Toward an ecological classification of soil bacteria. Ecology 2007; 88:1354-1364 10.1890/05-1839 17601128

[6] Campbell BJ Polson SW Hanson TE Mack MC Schuur EA The effect of nutrient deposition on bacterial communities in Arctic tundra soil. Environ Microbiol 2010; 12:1842-1854 10.1111/j.1462-2920.2010.02189.x 20236166

[7] Chu H Fierer N Lauber CL Caporaso JG Knight R Grogan P Soil bacterial diversity in the Arctic is not fundamentally different from that found in other biomes. Environ Microbiol 2010; 12:2998-3006 10.1111/j.1462-2920.2010.02277.x 20561020

[8] Pankratov TA Dedysh SN *Granulicella paludicola* gen. nov., sp. nov., *Granulicella pectinivorans* sp. nov., *Granulicella aggregans* sp. nov. and *Granulicella rosea* sp. nov., acidophilic, polymer degrading acidobacteria from Sphagnum peat bogs. Int J Syst Evol Microbiol 2010; 60:2951-2959 10.1099/ijs.0.021824-0 20118293

[9] Kishimoto N Kosako Y Tano T *Acidobacterium capsulatum* gen. nov., sp. nov.: an acidophilic chemoorganotrophic bacterium containing menaquinone from acidic mineral environment. Curr Microbiol 1991; 22:1-7 10.1007/BF02106205 23835745

[10] Eichorst SA Breznak JA Schmidt TM Isolation and characterization of soil bacteria that define *Terriglobus* gen. nov., in the phylum *Acidobacteria.* Appl Environ Microbiol 2007; 73:2708-2717 10.1128/AEM.02140-06 17293520 PMC1855589

[11] Männistö MK Rawat SR Starovoytov V Häggblom MM *Terriglobus saanensis* sp. nov., an acidobacterium isolated from tundra soil. Int J Syst Evol Microbiol 2011; 61:1823-1828 10.1099/ijs.0.026005-0 21186292

[12] Koch IH Gich F Dunfield PF Overmann J *Edaphobacter modestus* gen. nov., sp. nov., and *Edaphobacter aggregans* sp. nov., acidobacteria isolated from alpine and forest soils. Int J Syst Evol Microbiol 2008; 58:1114-1122 10.1099/ijs.0.65303-0 18450699

[13] Okamura K Kawai A Yamada T Hiraishi A *Acidipila rosea* gen. nov.,sp nov., an acidophilic chemoorganotrophic bacterium belonging to the phylum *Acidobacteria.* FEMS Microbiol Lett 2011; 317:138-142 10.1111/j.1574-6968.2011.02224.x 21255071

[14] Pankratov TA Kirsanova LA Kaparullina EN Kevbrin VV Dedysh SN *Telmatobacter bradus* gen. nov., sp. nov., a cellulolytic facultative anaerobe from subdivision 1 of the Acidobacteria and emended description of *Acidobacterium capsulatum* Kishimoto et al. Int J Syst Evol Microbiol 2012; 62:430-437 10.1099/ijs.0.029629-0 21460138

[15] Kulichevskaya IS Kostina LA Valásková V Rijpstra IC Sinninghe Damsté JS de Boer W Dedysh SN *Acidicapsa borealis* gen. nov., sp. nov. and *A. ligni* sp. nov., two novel subdivision 1 *Acidobacteria* from sphagnum peat and decaying wood. Int J Syst Evol Microbiol 2012; 62:1512-1520 10.1099/ijs.0.034819-0 21856984

[16] Dedysh SN Kulichevskaya IS Serkebaeva YM Mityaeva MA Sorokin VV Suzina NE Rijpstra WI Damste JS *Bryocella elongata* gen. nov., sp. nov., a novel member of Subdivision 1 of the *Acidobacteria* isolated from a methanotrophic enrichment culture, and emended description of *Edaphobacter aggregans* Koch et al. 2008. Int J Syst Evol Microbiol 2012; 62:654-664 10.1099/ijs.0.031898-0 21551329

[17] Kulichevskaya IS Suzina NE Liesack W Dedysh SN *Bryobacter aggregatus* gen. nov., sp. nov., a peat-inhabiting, aerobic chemoorganotroph from subdivision 3 of the *Acidobacteria.* Int J Syst Evol Microbiol 2010; 60:301-306 10.1099/ijs.0.013250-0 19651730

[18] Foesel BU Rohde M Overmann J *Blastocatella fastidiosa* gen. nov., sp. nov., isolated from semiarid savanna soil – The first described species of *Acidobacteria* subdivision 4. Syst Appl Microbiol 2013; 36:82-89 10.1016/j.syapm.2012.11.002 23266188

[19] Izumi H Nunoura T Miyazaki M Mino S Toki T Takai K Sako Y Sawabe T Nakagawa S *Thermotomaculum hydrothermale* gen. nov., sp. nov., a novel heterotrophic thermophile within the phylum *Acidobacteria* from a deep-sea hydrothermal vent chimney in the Southern Okinawa Trough. Extremophiles 2012; 16:245-253 10.1007/s00792-011-0425-9 22212657

[20] Liesack W Bak F Kreft JU Stackebrandt E *Holophaga foetida* gen.nov., sp. nov., a new homoacetogenic bacterium degrading methoxylated aromatic compounds. Arch Microbiol 1994; 162:85-90 10.1007/BF00264378 8085918

[21] Coates JD Ellis DJ Gaw CV Lovley DR *Geothrix fermentans* gen. nov., sp. nov., a novel Fe(III)-reducing bacterium from a hydrocarbon contaminated aquifer. Int J Syst Bacteriol 1999; 49:1615-1622 10.1099/00207713-49-4-1615 10555343

[22] Fukunaga Y Kurahashi M Yanagi K Yokota A Harayama S *Acanthopleuribacter pedis* gen. nov., sp. nov., a marine bacterium isolated from a chiton, and description of *Acanthopleuribacteraceae* fam. nov., *Acanthopleuribacterales* ord. nov., *Holophagales* ord. nov. and Holophagae classis nov. in the phylum ‘Acidobacteria’. Int J Syst Evol Microbiol 2008; 58:2597-2601 10.1099/ijs.0.65589-0 18984699

[23] Losey NA, Stevenson BS, Busse HJ, Damste JSS, Rijpstra WIC, Rudd S, Lawson PA. *Thermoanaerobaculum aquaticum* gen. nov., sp. nov., the first cultivated member of *Acidobacteria* subdivision 23, isolated from a hot spring. [PMID: 23771620]. [DOI 10.1099/ijs.0.051425-0] *Int J Syst Evol Microbiol* 2013; **63**:4149-4157. 23771620

[24] Ward NL Challacombe JF Janssen PH Henrissat B Coutinho PM Wu M Xie G Haft DH Sait M Badger J Three genomes from the phylum *Acidobacteria* provide insight into the lifestyles of these microorganisms in soils. Appl Environ Microbiol 2009; 75:2046-2056 10.1128/AEM.02294-08 19201974 PMC2663196

[25] Bryant DA Amaya M Garcia Costas AMG, Maresca JA, Chew AGM, Klatt CG, Bateson MM, Tallon LJ, Hostetler J, Nelson WC, Heidelberg JF, Ward DM. Candidatus *Chloracidobacterium thermophilum*: an aerobic phototrophic acidobacterium. Science 2007; 317:523-526 10.1126/science.1143236 17656724

[26] Männistö MK Tiirola M Häggblom MM Microbial communities in Arctic fjelds of Finnish Lapland are stable but highly pH dependent. FEMS Microbiol Ecol 2007; 59:452-465 10.1111/j.1574-6941.2006.00232.x 17328122

[27] Sait M Davis KE Janssen PH Effect of pH on isolation and distribution of members of subdivision 1 of the phylum *Acidobacteria* occurring in soil. Appl Environ Microbiol 2006; 72:1852-1857 10.1128/AEM.72.3.1852-1857.2006 16517631 PMC1393200

[28] Eichorst SA Kuske CR Schmidt TM Influence of plant polymers on the distribution and cultivation of bacteria in the phylum *Acidobacteria.* Appl Environ Microbiol 2011; 77:586-596 10.1128/AEM.01080-10 21097594 PMC3020536

[29] Rawat SR Männistö MK Bromberg Y Häggblom MM Comparative genomic and physiological analysis provides insights into the role of *Acidobacteria* in organic carbon utilization in Arctic tundra soils. FEMS Microbiol Ecol 2012; 82:341-355 10.1111/j.1574-6941.2012.01381.x 22486608

[30] Rawat SR Männistö MK Starovoytov V Goodwin L Nolan M Hauser L Land M Davenport KW Woyke T Häggblom MM Complete genome sequence of *Terriglobus saanensis* strain SP1PR4T, an *Acidobacteria* from tundra soil. Stand Genomic Sci 2012; 7:59-69 10.4056/sigs.3036810 23450133 PMC3570800

[31] Männistö MK Tiirola M Häggblom MM Effect of freeze-thaw cycles on bacterial communities of Arctic tundra soil. Microb Ecol 2009; 58:621-631 10.1007/s00248-009-9516-x 19367430

[32] Männistö MK Kurhela E Tiirola M Häggblom MM *Acidobacteria* dominate the active bacterial communities of sub-Arctic tundra with widely divergent winter-time snow accumulation and soil temperatures. FEMS Microbiol Ecol 2013; 84:47-59 10.1111/1574-6941.12035 23106413

[33] Woese CR Kandler O Wheelis ML Towards a natural system of organisms: proposal for the do-mains Archaea, Bacteria, and Eucarya. Proc Natl Acad Sci USA 1990; 87:4576-4579 10.1073/pnas.87.12.4576 2112744 PMC54159

[34] Thrash JC, Coates JD. Phylum XVII. *Acidobacteria* phyl. nov. In: Krieg NR, Staley JT, Brown DR, Hedlund BP, Paster BJ, Ward NL, Ludwig W, Whitman WB (eds), Bergey's Manual of Systematic Bacteriology, Second Edition, Volume 4, Springer, New York, 2011, p. 725.

[35] Validation List No 143. Int J Syst Evol Microbiol 2012; 62:1-4 10.1099/ijs.0.039487-0

[36] Cavalier-Smith T The neomuran origin of archaebacteria, the negibacterial root of the universal tree and bacterial megaclassification. Int J Syst Evol Microbiol 2002; 52:7-76 11837318 10.1099/00207713-52-1-7

[37] Judicial Commission of the International Committee on Systematics of Prokaryotes The nomenclatural types of the orders *Acholeplasmatales, Halanaerobiales, Halobacteriales, Methanobacteriales, Methanococcales, Methanomicrobiales, Planctomycetales, Prochlorales, Sulfolobales, Thermococcales, Thermoproteales* and *Verrucomicrobiales* are the genera *Acholeplasma, Halanaerobium, Halobacterium, Methanobacterium, Methanococcus, Methanomicrobium, Planctomyces, Prochloron, Sulfolobus, Thermococcus, Thermoproteus* and *Verrucomicrobium*, respectively. Opinion 79. Int J Syst Evol Microbiol 2005; 55:517-518 10.1099/ijs.0.63548-0 15653928

[38] Ludwig W, Euzeby J, Whitman WG. Draft taxonomic outline of the *Bacteroidetes, Planctomycetes, Chlamydiae, Spirochaetes, Fibrobacteres, Fusobacteria, Acidobacteria, Verrucomicrobia, Dictyoglomi,* and *Gemmatimonadetes* http://www.bergeys.org/outlines/Bergeys_Vol_4_Outline.pdf Taxonomic Outline 2008.

[39] Thrash JC, Coates JD. Family I. Acidobacteriaceae fam. nov. In: Krieg NR, Staley JT, Brown DR, Hedlund BP, Paster BJ, Ward NL, Ludwig W, Whitman WB (eds), Bergey's Manual of Systematic Bacteriology, Second Edition, Volume 4, Springer, New York, 2011, p. 728.

[40] Pankratov TA Dedysh SN *Granulicella paludicola* gen. nov., sp. nov., *Granulicella pectinivorans* sp. nov., *Granulicella aggregans* sp. nov. and *Granulicella rosea* sp. nov., acidophilic, polymer-degrading acidobacteria from Sphagnum peat bogs. Int J Syst Evol Microbiol 2010; 60:2951-2959 10.1099/ijs.0.021824-0 20118293

[41] Ashburner M Ball CA Blake JA Botstein D Butler H Cherry JM Davis AP Dolinski K Dwight SS Eppig JT Gene ontology: tool for the unification of biology. The Gene Ontology Consortium. Nat Genet 2000; 25:25-29 10.1038/75556 10802651 PMC3037419

[42] Tamura K Peterson D Peterson N Stecher G Nei M Kumar S MEGA5: molecular evolutionary genetics analysis using maximum likelihood, evolutionary distance, and maximum parsimony methods. Mol Biol Evol 2011; 28:2731-2739 10.1093/molbev/msr121 21546353 PMC3203626

[43] Liolios K Mavromatis K Tavernarakis N Kyrpides NC The Genomes On Line Database (GOLD) in 2007: status of genomic and metagenomic projects and their associated metadata. Nucleic Acids Res 2007; 36:D475-D479 10.1093/nar/gkm884 17981842 PMC2238992

[44] Field D Garrity G Gray T Morrison N Selengut J Sterk P Tatusova T Thomson N Allen MJ Angiuoli SV The minimum information about a genome sequence (MIGS) specification. Nat Biotechnol 2008; 26:541-547 10.1038/nbt1360 18464787 PMC2409278

[45] DOE Joint Genome Institute http://www.jgi.doe.gov/

[46] Bennett S Solexa Ltd. Pharmacogenomics 2004; 5:433-438 10.1517/14622416.5.4.433 15165179

[47] Margulies M Egholm M Altman WE Genome sequencing in microfabricated high-density picolitre reactors. Nature 2005; 437:376-380 16056220 10.1038/nature03959 PMC1464427

[48] Zerbino DR Birney E Velvet: algorithms for de novo short read assembly using de Bruijn graphs. Genome Res 2008; 18:821-829 10.1101/gr.074492.107 18349386 PMC2336801

[49] Ewing B Hillier L Wendl MC Green P Base-calling of automated sequencer traces using phred. I. Accuracy assessment. Genome Res 1998; 8:175-185 10.1101/gr.8.3.175 9521921

[50] Gordon D Abajian C Green P Consed: a graphical tool for sequence finishing. Genome Res 1998; 8:195-202 10.1101/gr.8.3.195 9521923

[51] The Phred/Phrap/Consed software package. http://www.phrap.com

[52] Han CS, Chain P. Finishing repeat regions automatically with Dupfinisher CSREA Press. In: Arabnia AR, Valafar H, editors. Proceedings of the 2006 international conference on bioinformatics & computational biology; 2006; June 26-29. CSREA Press. p 141-146.

[53] Hyatt D Chen GL Locascio PF Land ML Larimer FW Hauser LJ Prodigal: prokaryotic gene recognition and translation initiation site identification. BMC Bioinformatics 2010; 11:119 10.1186/1471-2105-11-119 20211023 PMC2848648

[54] Pati A Ivanova NN Mikhailova N Ovchinnikova G Hooper SD Lykidis A Kyrpides NC GenePRIMP: a gene prediction improvement pipeline for prokaryotic genomes. Nat Methods 2010; 7:455-457 10.1038/nmeth.1457 20436475

[55] Tatusov RL Koonin EV Lipman DJ A genomic perspective on protein families. Science 1997; 278:631-637 10.1126/science.278.5338.631 9381173

[56] Clusters of Orthologous Groups http://www.ncbi.nlm.nih.gov/COG

[57] Lowe TM Eddy SR tRNAscan-SE: a program for improved detection of transfer RNA genes in genomic sequence. Nucleic Acids Res 1997; 25:955-964 9023104 10.1093/nar/25.5.955 PMC146525

[58] Lagesen K Hallin P Rodland EA Staerfeldt HH Rognes T Ussery DW RNAmmer: consistent and rapid annotation of ribosomal RNA genes. Nucleic Acids Res 2007; 35:3100-3108 10.1093/nar/gkm160 17452365 PMC1888812

[59] Griffiths-Jones S Bateman A Marshall M Khanna A Eddy SR Rfam: an RNA family database. Nucleic Acids Res 2003; 31:439-441 10.1093/nar/gkg006 12520045 PMC165453

[60] Krogh A Larsson B von Heijne G Sonnhammer EL Predicting transmembrane protein topology with a hidden Markov model: application to complete genomes. J Mol Biol 2001; 305:567-580 10.1006/jmbi.2000.4315 11152613

[61] Bendtsen JD Nielsen H von Heijne G Brunak S Improved prediction of signal peptides: SignalP 3.0. J Mol Biol 2004; 340:783-795 10.1016/j.jmb.2004.05.028 15223320

[62] Markowitz VM Mavromatis K Ivanova N Chen IM Chu K Kyrpides N Expert Review of Functional Annotations for Microbial Genomes. Bioinformatics 2009; 25:2271-2278 10.1093/bioinformatics/btp393 19561336

[63] Cantarel BL Coutinho PM Rancurel C Bernard T Lombard V Henrissat B The Carbohydrate-Active EnZymes database (CAZy): an expert resource for Glycogenomics. Nucleic Acids Res 2009; 37:D233-D238 10.1093/nar/gkn663 18838391 PMC2686590

[64] Lombard V, Ramulu HG, Drula E, Coutinho PM and Henrissat B. The carbohydrate-active enzymes database (CAZy) in 2013. Nucleic Acids Research 1–6. 10.1093/nar/gkt1178 PMC3965031 24270786

